# How Does Perfectionism Influence the Development of Psychological Strengths and Difficulties in Children?

**DOI:** 10.3390/ijerph17114081

**Published:** 2020-06-08

**Authors:** Silvia Melero, Alexandra Morales, José Pedro Espada, Iván Fernández-Martínez, Mireia Orgilés

**Affiliations:** Department of Health Psychology, Miguel Hernández University, 03202 Elche, Spain; alexandra.moraless@goumh.umh.es (A.M.); jpespada@umh.es (J.P.E.); i.fernandez@umh.es (I.F.-M.); morgiles@umh.es (M.O.)

**Keywords:** child perfectionism, psychological difficulties, prosocial behavior, gender, age

## Abstract

This study aimed to examine differences in gender, age, and psychopathology, according to the perfectionism level, and to analyze how perfectionism dimensions contribute to the development of psychological strengths and difficulties in children. Participants were 319 Spanish students (52.4% girls) between 7 and 11 years old (*M* = 9.38, *SD* = 1.15). Children completed self-reported measures of perfectionism and psychological strengths and difficulties. The sample was divided into groups based on the perfectionism level (high, medium, and low). A one-way ANOVA (Analysis of variance), t-test, Pearson correlations, and 3-step hierarchical regression analyses were run. Results showed that 27.6% of the children belonged to the high perfectionism group, characterized by an elevated Socially Prescribed Perfectionism (SPP). Compared to girls, boys presented higher scores in all perfectionism measures. The younger children presented higher SPP and lower Self-oriented Perfectionism-Critical (SOP-Critical) than the older group. High perfectionism was related to psychological problems. The SOP-Critical increased the likelihood of developing emotional symptoms and total difficulties, and SPP was associated with behavioral and peer problems. In contrast, Self-oriented Perfectionism-Striving (SOP-Striving) was related to greater prosocial behavior. This research has important implications for the design of transdiagnostic strategies targeting the prevention and intervention of psychological difficulties in schoolchildren.

## 1. Introduction

Perfectionism is a personality trait characterized by the search for faultlessness and the establishment of very high levels of performance along with excessively critical self-evaluations [1,2,3]. This integrative definition encompasses a set of very strict self-imposed demands about what individuals believe they should become [4,5]. Other definitions have noted that perfectionism is a multi-dimensional concept composed of both intrapersonal and interpersonal traits [6]. For instance, Hewitt et al. [7] identified three main dimensions of perfectionism: self-oriented perfectionism, which involves self-requirements to be perfect, other-oriented perfectionism, which concerns demands for others to be perfect, and socially prescribed perfectionism, which involves perceptions that others require the self to be perfect. Other studies underline that perfectionism is composed of two traits: perfectionistic strivings and perfectionistic concerns [8,9,10]. Perfectionistic strivings have been suggested to be related to adaptive outcomes, such as positive affect, motivational orientations, hopes of success, etc., but perfectionistic concerns have been associated with maladaptive outcomes [8,9,10,11].

Although this pursuit of excellence has its adaptive facet, many studies have linked perfectionism to psychopathology, concluding that perfectionism is a transdiagnostic risk and a maintaining factor for multiple psychological disorders [10,11,12,13,14]. From the transdiagnostic perspective, perfectionism is a common psychological process in different disorders such as anxiety disorders, depression, obsessive-compulsive disorder (OCD), and eating disorders, and also favors co-occurrence among them [11]. Research on how perfectionism and its dimensions affect the field of psychopathology has increased in recent years. However, most of the investigations have been conducted with adolescent and adult populations [13,15,16]. Perfectionism in children has not been sufficiently explored in spite of being a critical stage for the development of perfectionism traits [1,5].

Children develop perfectionist traits through the interaction between their personal characteristics and their social environment’s demands [17]. Therefore, children set high standards of excellence on themselves according to their school and family environment demands and selective reinforcement of their achievements by adults [18,19]. Consequently, many multidimensional measures of perfectionism in children do not include the other-oriented perfectionism dimension since, according to the developmental perspective, children are not prepared to demand excellence from others [4,18,20,21]. The most widely used and supported of these is the Child and Adolescent Perfectionism Scale (CAPS), which conceptualizes child perfectionism based on two dimensions: Socially Prescribed Perfectionism (SPP) and Self-oriented perfectionism. Socially Prescribed Perfectionism includes an interpersonal component referring to environment demands. Self-oriented perfectionism (SOP) involves an intrapersonal dimension that implies the motivation to be perfectionist and self-critical [20,21]. Recent research has proposed a three-factorial model composed of Socially Prescribed Perfectionism (SPP) and two dimensions of SOP, which include Self-oriented perfectionism-Striving (SOP-Striving) and Self-oriented perfectionism-Critical (SOP-Critical) [22]. The SOP-Striving and SOP-Critical dimensions represent, respectively, the adaptive and maladaptive facets of SOP, as SOP-Striving showed positive correlations with parental expectations and academic achievement. SOP-Critical was positively associated with negative affect, parental criticism, anxiety, and depression [9,10,22]. These three dimensions are related to each other, showing small correlations between SOP-Critical and SOP-Strivings but moderate correlations between these two dimensions and SPP [22].

Numerous studies have examined the level of perfectionism in terms of gender and age. In general, results have shown that boys have a higher level of perfectionism than girls [20,21,23,24]. When distinguishing among dimensions, some studies found that males scored higher than females in intrapersonal perfectionism (especially SOP-Critical), that is, boys have higher standards of excellence, but the gender differences were not significant when concerning interpersonal perfectionism (SPP). This indicates that both perceive similar levels of perfectionist demands from their environment [18,22,24,25]. Regarding age, some studies suggest that perfectionism is higher at younger ages, especially socially prescribed perfectionism, due to greater parental demands in the first years of primary education [18,22,23]. However, the literature indicates that no significant differences by age have been found since perfectionism is a stable personality trait over time [24,26,27,28].

In relation to psychological problems, perfectionist children, who are constantly dealing with both internal and external pressures, show feelings of sadness, anger, futility, distress, embarrassment, a negative self-concept, dissatisfaction with themselves, and blame [12,29]. Research has reported that child maladaptive dimensions of perfectionism are related to psychopathological disorders such as depression [7,14,15,29,30], anxiety disorders [7,12,14,31], obsessive-compulsive disorder [32], and eating disorders [33], among others. If we consider the perfectionism dimensions separately, research reveals that SOP (specially SOP-Critical) interacts with social stress to predict anxiety and with achievement stress and social stress to predict depression in children [6,7,30]. Unlike adults, SPP in children is associated with both positive and negative affectivity, yet significant correlations have been found with anxiety, depression, social stress, anger, and interpersonal hostility [7,34,35]. Thus, both dimensions of perfectionism were associated with psychopathology outcomes across studies. Although research on child perfectionism has focused on its negative aspects, some studies have also found positive relationships between self-oriented perfectionism and psychosocial variables such as social support, empathy, and other prosocial behaviors [13,35].

The influence of perfectionism on children’s psychological difficulties and strengths has been scarcely explored. Therefore, the present study aimed to: 1) describe the level of perfectionism in a sample of Spanish children, 2) examine differences in perfectionism by gender and age, 3) analyze differences in emotional symptoms, behavioral problems, hyperactivity, peer problems, total difficulties, and prosocial behavior based on the perfectionism level, and 4) predict psychological strengths and difficulties from the perfectionism dimensions. Based on the previous literature [21,23,24,25], we hypothesize that gender differences will be found, but not by age. Furthermore, we expect children with high perfectionism to show greater psychological difficulties and lower prosocial behavior than low perfectionism children [7,14,30,35]. Lastly, we consider the SOP-Critical and SPP dimensions to be the most predictive of psychological difficulties, especially emotional symptoms and behavioral problems, respectively [7,13,27,35].

## 2. Materials and Methods

### 2.1. Participants

The study involved an incidental sample of Spanish-born children. The recruited sample consisted of 319 students between 7 and 11 years old enrolled between 3rd and 6th grade of a primary school in the province of Alicante, Spain. Half of them (*n* = 167, 52.4%) were girls and the mean age was 9.38 years (*SD* = 1.15).

A power analysis performed using G*Power 3.1.7. program [36] showed that, when one-way ANOVA (fixed effects, omnibus, one-way) is run, a minimum of 159 participants will be required to achieve a size effect of at least (f) 0.25 and power of 80%, based on VanVoorhis and Morgan [37] that recommended an alpha of 0.05 when there are three groups (high, medium, and low perfectionism). Based on the previously mentioned criteria, 88 children met the criteria for high perfectionism and 75 children for low perfectionism. A group of medium perfectionism group included 156 individuals.

### 2.2. Measures

The Child and Adolescent Perfectionism Scale (CAPS-S) [20] is a self-reported measure of perfectionism in youth. The Spanish version adapted by Vicent et al. [22] was used in this study. Its 13 items are structured in three factors: Self-oriented perfectionism-Striving (SOP-Striving), Self-oriented perfectionism-Critical (SOP-Critical), and Socially Prescribed Perfectionism (SPP). Items are rated on a five-point scale ranging from 1 (false—not at all true of me) to 3 (very true of me), and higher scores reflect greater perfectionism. The reliability was adequate in both the original scale [21], composed of only two dimensions (SOP = 0.85, SPP = 0.81), and the Spanish version made up of three subscales (SOP-Striving = 0.74, SOP-Critical = 0.73, SPP = 0.80) [22]. In this study, ordinal alphas were α = 0.82 for the total CAPS-S, α = 0.68 for SOP-Striving, α = 0.71 for SOP-Critical, and α = 0.82 for SPP.

The Strengths and Difficulties Questionnaire (SDQ-C) [38] is a tool designed to assess prosocial behavior and general difficulties in children and adolescents. It consists of 25 items divided into five subscales of five items each: emotional symptoms, conduct problems, hyperactivity/inattention, peer problems, and prosocial behavior. Children should rate each item on a 3-point scale ranging from 0 (not true) to 2 (certainly true). The total difficulties score is obtained by summing all subscales, except for the prosocial behavior subscale (scores range from 0 to 40). The SDQ-C Spanish version has shown adequate reliability for the total difficulties score (α = 0.84) and its subscales (range from α = 0.71–0.75) [39]. Ordinal alphas of the SDQ-C subscales ranged from α = 0.55 to 0.78 in the current study.

### 2.3. Procedure

This study was approved by the Ethics Committee of the Miguel Hernández University, Spain (DPS.MO.01.17). The sample was recruited from a school in the province of Alicante and selected for convenience, according to the accessibility criteria and socioeconomic representativeness of the Spanish population. A meeting was held with the school principal to explain the objectives and procedure of the study and to request their participation. The school principal distributed a letter to parents of children in 3rd grade to 6th grade inviting them to participate voluntarily and providing information about the study and the confidentiality of their data. Parental informed consent was obtained for the children whose families agreed with the study.

Children who participated were gathered in three classrooms at their school and were requested to provide their socio-demographic data in writing and completing the CAPS-S and SDQ-C self-report measures during school hours. Data were collected via paper-and-pencil questionnaires under the supervision of some researchers in charge of the study. Supervisors read the items aloud and assisted with questions, while the students completed them individually. Children were informed that the data would be treated confidentially, and their participation was voluntary.

### 2.4. Data Analyses

Descriptive statistics were used to describe the sample. According to the study by Vicent et al. [34], the variable CAPS-S (total perfectionism) was dichotomized in high scores (above 75th percentile) and low scores (under 25th percentile). The rest of the participants were considered in the medium level of the perfectionism group. Differences in sociodemographic variables, CAPS-S items, and strengths and difficulties (SDQ-C subscales) were analyzed based on the level of perfectionism (high, medium, and low groups) using a cross-table for categorical variables. One-way ANOVA (Analysis of variance) for continuous variables were performed. Adjusted residuals were calculated in the cross-table to identify differences among groups, when the test was statistically significant [40]. Bonferroni correction applied to *p*-values was used to reduce the risk of Type I errors post hoc analysis. Bonferroni post hoc tests were run when differences were statistically significant. Partial eta squared (η_p_^2^) was calculated as the effect size, where 0.009 constitutes a small effect, 0.058 shows a medium effect, and 0.137 shows a large effect, according to Cohen’s criteria [41]. Cramer’s *V* was calculated as a measure of association between two categorical variables, and it was interpreted as follows: >0.25 very strong, >0.15 strong, >0.10 moderate, >0.05 weak, and >0 no or very weak [42].

Differences by gender (boys and girls) and age (7–9 years old and 10–11 years old groups) were analyzed using independent sample *t*-tests. Cohen’s *d* was calculated to report the effect size for statistically significant differences, where 0.20 is considered small, 0.50 is considered medium, and 0.80 is considered large [41]. Pearson correlations were calculated to analyze the relationship between continuous variables, and the results. The skew and kurtosis were acceptable for continuous variables. To test the association between strengths and difficulties and perfectionism, six separate 3-step hierarchical regression analyses were run with emotional problems, conduct problems, hyperactivity, peer problems, prosocial behavior, and total difficulties as dependent variables. Gender and age were included in the first step as covariates because differences in perfectionism by these variables were identified previously. SOP-Striving, SOP-Critical, and SPP were included in the second step as independent variables. Interactions between variables were analyzed in step 3. Continuous variables were mean-centered to avoid multicollinearity. All analyses were performed using SPSS v.26. The figure was plotted using the ggplot2 package [43] in the framework of R 3.5.2 with *R* Studio 1.1.453 [44].

## 3. Results

### 3.1. Descriptive Data of the Sample

We begin by presenting descriptive data of the sample and by perfectionism (Table 1). Differences in age (by year) and school level among the three levels of perfectionism (high, medium, and low) were observed. The associations between perfectionism (3 levels) and age and school level, respectively, were moderate (Cramer’s *V* = 0.15). Pairwise cross-tabulations indicated differences in the proportion of participants in each category of participant’s age (χ^2^ = 13.94, *p* = 0.007, Cramer’s *V* = 0.007) and, consequently, during the school year as well (χ^2^ = 12.93, *p* = 0.005, Cramer’s *V* = 0.005). A higher proportion of children in the high perfectionism were in the 11-year-old category, compared to the low perfectionism group (29.5% vs. 14.7%), and, consequently, were enrolled in the sixth school year category compared to the low perfectionism group (31.8% vs. 8.6%). These associations were not significant after the traditional Bonferroni correction (*p* < 0.003 for children’s age and *p* < 0.004 for the school level). Hence, it is concluded that there was not a statistically significant relationship between children’s age and perfectionism, and between school level and perfectionism. The three groups were equivalent in participants’ gender and mean age, nationality, and number of siblings.

### 3.2. Differences in CAPS-S by Perfectionism Level

Table 2 presents the scores of CAPS-S items and subscales (SOP-Striving, SOP-Critical, SPP, and CAPS-S) as a function of the perfectionism level. Regarding the total perfectionism score, the highest scores were obtained in the SOP-Striving subscale. Examining these outcomes by level of perfectionism, the most perfectionist children scored higher on SPP, while the least perfectionist children scored higher on SOP-Striving. The SOP-Critical subscale was the lowest for all levels of perfectionism.

In the SOP-Striving subscale, the item with the highest scores for the three perfectionism groups was “I always try for the top score on a test” (Item 6) followed by “I feel that I have to do my best all the time” (Item 4). In addition, the lowest-scoring item at all levels was “I want to be the best at everything I do” (Item 2). As for the SOP-Critical subscale, perfectionist children in any group scored higher on the item “I get mad at myself when I make a mistake” (Item 11). Regarding the differences between groups, the least scored item for children in the medium and high perfectionism groups was “I get upset if there is even one mistake in my work” (Item 14), while the lowest-rated item for the low perfectionism group was “I get upset if there is even one mistake in my work” (Item 22).

Greater heterogeneity was found in the SPP dimension among the three levels of perfectionism. While item “There are people in my life who expect me to be perfect” (Item 5) was the highest for the high perfectionism group, which was followed by “My family expects me to be perfect” (Item 8), the item “My teachers expect my work to be perfect” (Item 17) was the highest for the medium and low perfectionism group. The items with the lowest scores were “People expect more from me than I am able to give” (Item 10) for the children with high perfectionism and “Other people always expect me to be perfect” (Item 13) for those with medium and low perfectionism.

### 3.3. Descriptive Data on Age and Gender in Perfectionism

Table 3 indicates differences in perfectionism measures (SOP-Striving, SOP-Critical, SPP, and CAPS-S) by gender (two groups: boys vs. girls) and age (two groups: 7 to 9 years vs. 10 to 11 years). Compared to girls, boys presented higher scores in all perfectionism measures. However, effect sizes were small (*d* = from 0.21 to 0.31). Children aged 7–9 years old presented lower scores in SOP-Critical, but higher in SPP, compared to the oldest group. Effect size coefficients were small (*d* = 0.29) and moderate (*d* = 0.49), respectively.

### 3.4. Descriptive Data on Strengths and Difficulties in Perfectionism

Table 4 shows differences in emotional symptoms, conduct problems, hyperactivity, peer problems, and prosocial behavior by the three levels of perfectionism (high, medium, and low). One-way ANOVA indicated differences in emotional problems and conducts among these three groups. Children in the high perfectionism group were more likely to present emotional problems, conduct problems, and total difficulties (only marginally significant) than those in the low perfectionism group. No differences were found in hyperactivity, peer problems, and prosocial behavior among the three levels of perfectionism.

### 3.5. Regression Analyses Predicting Child Strengths and Difficulties

**Figure 1:**
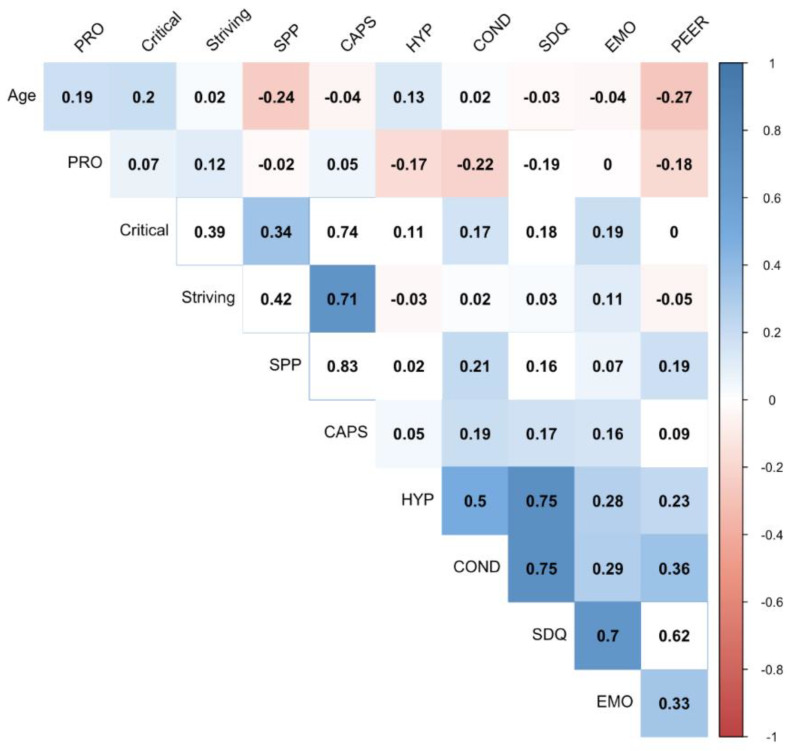
Pearson correlations among variables. Critical—Self-oriented Perfectionism-Critical, Striving—Self-oriented Perfectionism-Striving, SPP—Socially Prescribed Perfectionism, CAPS—Total Perfectionism Score, HYP—Hyperactivity/Inattention, COND—Conduct Problems, SDQ—Total Difficulties, EMO—Emotional Symptoms, PEER—Peer Problems, PRO—Prosocial Behavior.

Figure 1 presents the sample correlations between the variables included in the multiple hierarchical regression models. Correlations among SDQ-C subscales (emotional problems, conduct problems, hyperactivity, peer problems, and total difficulties) were direct and from small-to-high values (from α = 0.23 to 0.75). Prosocial behavior, as expected, was indirectly related to the rest of the SDQ-C subscales (from −0.18 to −0.22). Correlations among subscales of CAPS-S were direct and moderate (from 0.34 to 0.42) and the correlations between these subscales and the total score of the CAPS-S were direct and high (from 0.71 to 0.83). SOP-Critical was significantly directed to emotional problems, conduct problems, hyperactivity, and total difficulties. However, coefficients were low (from 0.11 to 0.19). SOP-Striving was significantly undirected to peer problems, but the correlation was low. SPP was significantly directed to behavioral problems, emotional problems, and total difficulties, and undirected to prosocial behavior in which these correlations were small (from −0.02 to 0.21). CAPS-S was significantly directed to behavioral problems, emotional problems, total difficulties, hyperactivity, and prosocial behavior (from 0.05 to 0.19).

Table 5 reports summary statistics for the three-step hierarchical regression models for emotional problems, conduct problems, hyperactivity, peer problems, prosocial behavior, and total difficulties. Gender and age were included in step 1, and perfectionism measures (SOP-Striving, SOP-Critical, and SPP) were added in step 2. Interactions between variables were analyzed in step 3. Table 5 reports step 3 when the interaction between terms for the model increased the explained variance.

#### 3.5.1. Emotional Problems

In step 1, gender and age were not significant and accounted for less than 1% of the variance of emotional problems. Adding the three perfectionism measures to the adjusted model in the second step accounted for 3% of the variance, *F*_(316)_ = 2.90, *p* < 0.01. The small contribution to the model was related to SOP-Critical. Children presenting higher levels of SOP-Critical were more likely to present emotional problems (*B* = 0.11, 95% CI = 0.05, 0.17, *p* < 0.001).

#### 3.5.2. Conduct Problems

In step 1, gender (but not age) was significant and accounted for 1% of the variance of conduct problems in the adjusted model. Boys were more likely to present higher scores in conduct problems. Adding the three perfectionism measures to the adjusted model in the second step accounted for 7% of the variance, *F*_(316)_ = 5.99, *p* < 0.001. The contribution to the model was related to gender, SOP-Striving, and SPP. Being a boy (compared to girls) (*B* = −0.50, 95% CI = −0.91, −0.08, *p* = 0.01), and having a lower score in SOP-Striving (*B* = −0.08, 95% CI = −0.16, −0.01, *p* < 0.02) and a higher score in SPP were associated with higher conduct problems (*B* = 0.08, 95% CI = 0.0.3, 0.12, *p* < 0.001). Step 3 is not reported in Table 5 because the addition of the interaction between gender and SOP-Striving to the model failed to increase the variance explained. Including the interaction between gender and SPP during step 3 also failed to increase the variance accounted for.

#### 3.5.3. Hyperactivity

In step 1, gender and age were significant and accounted for 3% of the variance of the hyperactivity adjusted model. Boys and older children were more likely to present higher scores in hyperactivity. Adding the three perfectionism measures to the adjusted model in the second step did not contribute toward increasing the explained variance of the model, *F*_(316)_ = 3.47, *p* = 0.004. SOP-Striving, SOP-Critical, and SPP measures were unrelated to hyperactivity symptoms.

#### 3.5.4. Peer Problems

In step 1, gender and age were significant and accounted for 9% of the variance of peer problems in the adjusted model. Boys and younger children were more likely to present higher scores in peer problems. Adding the three perfectionism measures to the adjusted model in the second step accounted for 10% of the variance, *F*_(313)_ = 8.60, *p* < 0.001. The contribution to the model was mostly related to gender, age, and slightly to SOP-Striving and SPP. Being a boy (compared to a girl) (*B* = −0.44, 95% CI = −0.77, −0.10, *p* = 0.01), being younger (*B* = −0.32, 95% CI = −0.47, −0.16, *p* < 0.001), having a lower score in SOP-Striving (*B* = −0.06, 95% CI = −0.12, −0.006, *p* = 0.03), and a higher score in SPP (*B* = 0.04, 95% CI = 0.01, 0.08, *p* = 0.01) were associated with higher peer problems. Step 3 is not reported in Table 5 because the addition of the interaction between gender and SOP-Striving, as well as the interaction between gender and SPP to the model, failed to increase the variance explained. Including the interaction between age and SOP-Striving as well as age and SPP at step 3 also failed to increase the variance accounted for.

#### 3.5.5. Prosocial Behavior

In step 1, gender and age were significant and accounted for 5% of the variance of the prosocial behavior adjusted model. Girls and older children were more likely to present prosocial behaviors. Adding the three perfectionism measures to the adjusted model in the second step accounted for 6% of the variance, *F*_(313)_ = 5.54, *p* < 0.001. The contribution to the model was mostly related to gender, and age, and slightly to SOP-Striving. Being a girl (compared to a boy) (*B* = 0.50, 95% CI = −0.16, 0.84, *p* = 0.004), being older (*B* = 0.23, 95% CI = 0.07, 0.39, *p* = 0.004), and having a higher score in SOP-Striving (*B* = 0.06, 95% CI = 0.005, 0.12, *p* = 0.03) were associated with greater prosocial behaviors. Lastly, adding the interaction between gender and SOP-Striving in step 3 resulted in another 1% of the variance being explained (7% in total), a significant increment in *R*^2^, *F*_(1,138)_ = 7.50, *p* < 0.001. However, including the interaction between age and SOP-Striving at step 3 failed to increase the variance accounted for.

#### 3.5.6. Total Difficulties

In step 1, gender (but not age) was significant, and accounted for 2% of the variance of total difficulties in the adjusted model. Boys were more likely to present total difficulties. Adding the three perfectionism measures to the adjusted model in the second step accounted for 5% of the variance, *F*_(318)_ = 4.49, *p* = 0.001. The contribution to the model was related to gender and SOP-Critical. Being a boy (compared to a girl) (*B* = −1.54, 95% CI = −0.2.81, −0.28, *p* = 0.01) and having a higher score in SOP-Critical (*B* = 0.22, 95% CI =0.05, 0.39, *p* = 0.01) were associated with greater total difficulties. Including the interaction between gender and SOP-Critical at step 3 failed to increase the variance accounted for.

## 4. Discussion

The goal of the present study consisted of evaluating the level of perfectionism in a community sample of Spanish children from 7 to 11 years old. In addition, possible gender and age differences in child perfectionism and the relationships between perfectionism domains and psychopathology were explored. As a novelty, the contribution of perfectionism in the children’s prosocial behavior was also analyzed.

Based on the multi-dimensional analysis of perfectionism, it was observed that the SOP-Striving was the most developed factor in the participants, which implies that commitment, effort, and self-demanding behaviors predominated, especially at the school level [25]. When the sample was divided into groups according to percentiles, about half of the children had a medium perfectionism level and more than a quarter belonged to the high perfectionism group. In this high perfectionism group, the most characteristic trait was the SPP, which confirmed that these children were more influenced by the expectations of others, specifically their family and teachers [18,19]. The SOP-Critical trait was the least prevalent in all three levels of perfectionism and was expressed mainly through anger at themselves for making mistakes [7,25,29]. Therefore, as in other similar studies, the adaptive facet of child perfectionism predominated in this sample [24,25].

The results supported our hypothesis showing significant differences in perfectionism, according to gender. In line with previous literature, males scored higher in the perfectionism measures than females [18,20,21,23,24]. Some studies that examined gender differences in each dimension found significant differences only in SOP [22,24], which showed that boys set elevated self-imposed standards when compared to girls, or only in SPP [20,21] in which boys reported higher requirements to be perfect from authority figures than girls. In contrast, our data showed that boys scored significantly higher in all perfectionism dimensions, which indicated that boys are more self-demanding, self-critical, and perceive a greater perfectionist demand in their environment than do girls [18,23]. Furthermore, the effect size of these differences was larger than in previous studies [24]. These gender differences may also be influenced by cultural and age factors, as international studies with adolescents and adult samples found no difference between men and women in perfectionism levels [45,46]. Future studies should address the relationship between perfectionism and these psychosocial variables [13].

In relation to age, it was hypothesized that no differences would be found between younger and older children. According to previous studies, no significant association was obtained between the level of perfectionism and age nor between perfectionism and the school level [24,26,28]. Regarding subscales, SPP was higher in the younger children (7–9 years) and SOP-Critical was higher in the older group (10–11 years). These results were also obtained in other studies conducted with children and adolescents where SPP decreased significantly over time [22,47] and SOP-Critical was higher in students enrolled in higher grades [18,22,23]. These findings may be explained because younger children perceive greater pressure to be perfectionists from adults. However, over time, these demands diminish and children set high standards for themselves and are more self-critical [18].

As expected, the presence of elevated levels of perfectionism was associated with psychological difficulties in children [7,11,14]. Specifically, these children were more likely to suffer from emotional and behavioral problems and adjustment difficulties than children with low perfectionism. Moreover, children in the medium perfectionism group showed a risk of emotional problems, which confirms the close relationship between internalizing symptoms and perfectionism [21,29,30]. In this study, both the SPP and SOP-Critical dimensions were significantly and directly related to emotional and behavioral problems, hyperactivity, and total difficulties. In contrast, the SOP-Striving was associated with fewer peer problems. These outcomes are consistent with previous research, which suggests that SOP-Critical corresponds to the maladaptive component and SOP-Striving to the adaptive component of intrapersonal perfectionism [9,10,22].

This study examined the interaction between perfectionism dimensions and children’s psychological strengths and difficulties. As previously hypothesized, the SOP-Critical was the most predictive perfectionism subscale for psychological difficulties in children, especially for boys [18,22,27]. Similarly, children with a higher level of self-critical perfectionism showed greater emotional symptoms as they tend to feel guilt, sadness, anger, and remorse when they make mistakes, which, again, highlighted the involvement of this dimension in the development of anxious and depressive symptomatology [14,15,29,30]. On the other hand, behavioral and peer problems were found to be more likely in boys with low SOP-Striving and high SPP. This suggests that children who have low standards of excellence, but perceive high demands on their environment, are more likely to exhibit hostility toward others and antisocial behaviors, likely due to their frustration by not meeting the others’ expectations [7,29,35].

Regarding psychological strengths, prosocial behavior was related to the SOP-Striving and SPP variables. On the one hand, prosocial behavior correlated indirectly with SPP, which indicated that behaviors such as empathy, kindness, helping others, and offering emotional support were lower in children with more socially prescribed perfectionism. On the other hand, it was found that older girls who scored high in SOP-Striving showed greater prosocial behaviors. These results are consistent with those obtained in the study of Stoeber et al. [35] in which the SOP displayed positive relationships with altruism, interest in others, affiliative humor, and prosocial goals. The SPP showed a consistent pattern of negative relationships with these prosocial behaviors.

Given the cross-sectional nature of the current study, some limitations should be noted when inferring conclusions from this research. The sample comprised children from a single school, which makes it difficult to generalize the results because of the risk of possible bias. Furthermore, data were collected through self-report measures. Thus, future research should include parent and teacher reports, or other assessment techniques such as interviewing. In addition, participants belonged to a community sample. Hence, current findings should be replicated in specific clinical samples [22]. Lastly, it is recommended to conduct longitudinal studies that support the relationships between perfectionism and children’s psychological strengths and difficulties [14].

## 5. Conclusions

Overall, the findings suggest that high perfectionism in children was related to psychological problems, especially the self-critical aspect of this construct, which supported previous literature about its maladaptive nature [6,10,13,14,27]. In addition, from our knowledge, the adaptive value of perfectionism in children’s psychological strengths is highlighted for the first time. Our results have important clinical and research implications and suggest that preventive interventions may be aimed primarily at reducing self-critical perfectionism, especially in older children, and perfectionism derived from environmental demands, as these dimensions are involved in the development, maintenance, and course of a wide range of psychopathological disorders. Special emphasis should also be placed on boys, as they may require more intensive and longer-lasting interventions because of their tendency to set high standards of excellence. Therefore, this study provides key information for the design of transdiagnostic strategies targeting the prevention and intervention of psychological difficulties in schoolchildren.

## Figures and Tables

**Table 1:** Demographic description of the participants.

			Perfectionism					
		Total	High (*n* = 88)	Medium (*n* = 156)	Low (*n* = 75)	*F*^3^/χ^2 4^	*p* ^5^	Cramer’s *V*
Female *N* (*%*)		167 (52.4)	46 (61.3)	80 (51.3)	41 (46.6)	3.66	0.16	-
Age (years) *M* ^1^ (*SD*) ^2^		9.38 (1.15)	9.42 (1.25)	9.31 (1.14)	9.45 (1.03)	0.45	0.63	-
Age (years) *N* (*%*)								
	7	10 (3.1)	2 (2.3)	6 (3.8)	2 (2.7)	15.81	0.04	0.15
	8	77 (24.1)	26 (29.5)	38 (24.4)	13 (17.3)			
	9	81 (25.4)	19 (21.6)	42 (26.9)	20 (26.7)			
	10	85 (26.6)	15 (17)	41 (26.3)	29 (38.7)			
	11	66 (20.7)	26 (29.5)	29 (18.6)	11 (14.7)			
Nationality								
	Spanish	311 (97.8)	86 (97.7)	151 (97.4)	74 (98.7)	0.36	0.83	
	Other	8 (2.2)	2 (2.3)	4 (2.6)	1 (1.3)			
Number of siblings (years) *M* ^1^ (*SD*) ^2^		1.01 (0.72)	1.05 (0.72)	0.97 (0.74)	1.05 (0.76)	0.49	0.61	-
Number of siblings *N* (*%*)								
	0	62 (19.4)	13 (14.8)	34 (21.8)	15 (20)	10.36	0.24	-
	1	209 (65.5)	63 (71.6)	101 (64.7)	45 (60)			
	2	35 (11)	7 (8)	16 (10.3)	12 (16)			
	3	9 (2.8)	5 (5.7)	2 (1.3)	2 (2.7)			
	4	4 (1.3)	0 (0)	3 (1.9)	1 (1.3)			
School level *N* (*%*)								
	3	75 (23.5)	27 (30.7)	36 (23.1)	12 (16)	14.83	0.02	0.15
	4	86 (27)	17 (19.3)	47 (30.1)	22 (29.3)			
	5	80 (25.1)	16 (18.2)	37 (23.7)	27 (36)			
	6	78 (24.5)	28 (31.8)	36 (23.1)	14 (18.7)			

^1.^ Mean. ^2^ Standard Deviant. ^3^ F of ANOVA. ^4^ Chi-square. ^5^ *p*-value.

**Table 2:** Mean (standard deviation) for CAPS-S items and differences in scores among the three groups: high, medium, and low perfectionism.

CAPS-S Items		Perfectionism			*F* ^1^	*p* ^2^	Post Hoc ^3^	Partial Eta Square
	Total	High (*n* = 88)	Medium (*n* = 156)	Low (*n* = 75)				
SOP-Striving ^7^								
1. I try to be perfect in everything I do.	3.61 (1.34)	4.36 (0.98)	3.75 (1.21)	2.45 (1.20)	57.67	≤0.001	H ^4^ > M ^5^ H > L ^6^ M > L	0.26
2. I want to be the best at everything I do.	2.97 (1.46)	4.14 (1.01)	2.93 (1.33)	1.66 (0.94)	91.49	≤0.001	H > M H > L M > L	0.36
4. I feel that I have to do my best all the time.	4.47 (0.95)	4.80 (0.58)	4.55 (0.86)	3.93 (1.23)	19.89	≤0.001	H > L M > L	0.11
6. I always try for the top score on a test.	4.67 (0.81)	4.95 (0.25)	4.71 (0.70)	4.26 (1.21)	16.17	≤0.001	H > L M > L	0.09
SOP-Critical ^8^								
11. I get mad at myself when I make a mistake.	3.34 (1.54)	4.19 (1.18)	3.33 (1.50)	2.37 (1.44)	33.73	≤0.001	H > M H > L M > L	0.17
14. I get upset if there is even one mistake in my work.	2.26 (1.51)	3.29 (1.62)	2.10 (1.38)	1.37 (0.78)	43.24	≤0.001	H > M H > L M > L	0.21
20. Even when I pass, I feel that I have failed if I didn’t get one of the highest marks in the class.	2.36 (1.56)	3.54 (1.56)	2.19 (1.41)	1.34 (0.79)	56.77	≤0.001	H > M H > L M > L	0.26
22. I cannot stand to be less than perfect.	2.37 (1.53)	3.52 (1.54)	2.22 (1.38)	1.33 (0.75)	57.93	≤0.001	H > M H > L M > L	0.26
SPP ^9^								
5. There are people in my life who expect me to be perfect.	3.18 (1.59)	4.34 (1.01)	3.20 (1.49)	1.78 (1.20)	76.79	≤0.001	H > M H > L M > L	0.32
8. My family expects me to be perfect.	2.92 (1.59)	4.22 (1.16)	2.86 (1.45)	1.50 (0.89)	93.81	≤0.001	H > M H > L M > L	0.37
10. People expect more from me than I am able to give.	3.06 (1.57)	3.96 (1.37)	2.94 (1.51)	2.28 (1.41)	28.35	≤0.001	H > M H > L M > L	0.15
13. Other people always expect me to be perfect.	2.72 (1.60)	4.09 (1.22)	2.59 (1.48)	1.37 (0.74)	92.95	≤0.001	H > M H > L M > L	0.37
17. My teachers expect my work to be perfect.	3.50 (1.43)	4.21 (1.06)	3.58 (1.34)	2.49 (1.42)	36.51	≤0.001	H > M H > L M > L	0.18
SOP-Striving (6–20)	15.73 (3.14)	18.27 (1.67)	15.95 (2.45)	12.32 (2.66)	134.47	≤0.001	H > M H > L M > L	0.46
SOP-Critical (4–20)	10.35 (4.31)	14.55 (3.57)	9.86 (3.33)	6.42 (2.09)	137.23	≤0.001	H > M H > L M > L	0.46
SPP (5–25)	15.40 (5.50)	20.84 (3.16)	15.19 (4.30)	9.44 (2.89)	191.04	≤0.001	H > M H > L M > L	0.54
CAPS-S ^10^ (19–65)	41.49 (10.01)	53.67 (4.44)	41.01 (4.02)	28.18 (4.22)	751.38	≤0.001	H > M H > L M > L	0.82

Note: The numbers are the correspondence of the CAPS-S scale with the original 22-item CAPS version of Flett et al. (2016). ^1^ F of ANOVA, ^2^ *p*-value, ^3^ Bonferroni correction applied to *p*-values was used to reduce the risk of type I errors post hoc analysis, ^4^ High, ^5^ Medium, ^6^ Low, ^7^ self-oriented perfectionism-Striving, ^8^ self-oriented perfectionism-Critical, ^9^ socially prescribed perfectionism, ^10^ total perfectionism score.

**Table 3:** Mean (Standard Deviation) for CAPS-S and its subscales according to gender and age.

	Gender					Age				
	Girls (*n* = 167)	Boys (*n* = 152)	*t*-test	*p ^5^*	*d ^6^*	7–9 years (*n* = 168)	10–11 years (*n* = 151)	*t*-test	*p ^5^*	*d ^6^*
SOP-Striving ^1^	15.41 (3.13)	16.09 (3.13)	1.91	0.05	0.21	15.73 (2.98)	15.74 (3.33)	−0.01	0.99	-
SOP-Critical ^2^	9.80 (4.10)	10.94 (4.46)	2.37	0.01	0.26	9.75 (4.24)	11.01 (4.30)	−2.65	0.008	0.29
SPP ^3^	14.78 (5.44)	16.07 (5.50)	2.10	0.03	0.23	16.66 (5.38)	13.99 (5.31)	4.45	0.001	0.49
CAPS-S ^4^	40.01 (9.85)	43.11 (9.97)	2.79	0.005	0.31	42.15 (9.45)	40.7 (10.58)	1.24	0.21	-

^1.^ Self-oriented Perfectionism-Striving. ^2^ Self-oriented Perfectionism-Critical. ^3^ Socially Prescribed Perfectionism. ^4^ Total Perfectionism Score. ^5^ *p*-value. ^6^ Cohen’s *d*.

**Table 4:** Mean (Standard Deviation) for SDQ-C subscales among the three groups: high, medium, and low perfectionism.

		Perfectionism			*F*	*p*	Post Hoc ^3^	Partial Eta Square
	Total	High (*n* = 88)	Medium (*n* = 156)	Low (*n* = 75)				
Emotional problems	2.71 (2.31)	2.96 (2.51)	2.85 (2.25)	2.10 (2.11)	3.45	0.03	H ^1^ > L ^2^	0.02
Conduct problems	2.41 (1.93)	2.75 (2.06)	2.44 (1.87)	1.93 (1.84)	3.72	0.02	H > L	0.02
Hyperactivity	4.12 (2.35)	4.05 (2.32)	4.25 (2.48)	3.92 (2.10)	0.56	0.57	-	-
Peer problems	1.49 (1.58)	1.55 (1.73)	1.49 (1.63)	1.44 (1.28)	0.11	0.89	-	-
Prosocial behavior	8.36 (1.57)	8.59 (1.57)	8.28 (1.65)	8.29 (1.37)	1.20	0.30	-	-
Total difficulties	10.74 (5.80)	11.32 (6.15)	11.05 (5.89)	9.40 (4.97)	2.71	0.06	-	-

^1.^ High. ^2^ Low. ^3^ Bonferroni correction applied to *p*-values was used to reduce the risk of type I errors post hoc analysis.

**Table 5:** Results from hierarchical regression examining the association between perfectionism and symptoms’ emotional symptoms, behavior, hyperactivity, peer problems, and prosocial behavior.

Predictor Variables	*B* ^2^	95% CI ^3^ (B)	*β* ^4^	*t* Statistic ^5^	*p*-Value ^6^	ΔR^2 7^	Adj.R^2 8^
DV ^1^: Emotional symptoms							
Step 1				0.25	0.77	0.002	−0.005
Constant	3.42	1.30, 5.53		3.18	0.002		
Gender	−0.08	−0.59, 0.43	−0.01	−0.30	0.75		
Age	−0.07	−0.29, 0.15	−0.03	−0.62	0.53		
Step 2				2.90	0.01	0.04	0.03
Constant	2.88	0.27, 5.48		2.17	0.03		
Gender	0.05	−0.45, 0.56	0.01	0.21	0.82		
Age	−0.18	−0.41, 0.05	−0.09	−1.49	0.13		
SOP-Striving ^9^	0.04	−0.05, 0.13	0.05	0.88	0.37		
SOP-Critical ^10^	0.11	0.05, 0.17	0.20	3.17	<0.001		
SPP ^11^	−0.01	−0.07, 0.03	−0.04	−0.69	0.49		
DV^1^: Conduct problems							
Step 1				3.96	0.02	0.02	0.01
Constant	2.33	0.59, 4.08		2.63	0.009		
Gender	−0.60	−1.02, −0.17	−0.15	−2.79	0.006		
Age	0.04	−0.14, 0.22	0.02	0.44	0.65		
Step 2				5.99	<0.001	0.08	0.07
Constant	1.27	−0.85, 3.40		1.17	0.24		
Gender	−0.50	−0.91, −0.08	−0.12	−2.36	0.01		
Age	0.10	−0.09, 0.29	0.06	1.04	0.29		
SOP-Striving ^9^	−0.08	−0.16, −0.01	−0.14	−2.25	0.02		
SOP-Critical ^10^	0.05	−0.005, 0.10	0.11	1.79	0.07		
SPP ^11^	0.08	0.03, 0.12	0.23	3.59	<0.001		
DV: Hyperactivity							
Step 1				6.51	0.002	0.04	0.03
Constant	1.82	−0.28, 3.92		1.70	0.09		
Gender	−0.69	−1.20, −0.18	−0.14	2.67	0.008		
Age	0.28	0.06, 0.50	0.14	2.51	0.01		
Step 2				3.47	0.004	0.05	0.03
Constant	2.34	−0.29, 4.97		1.74	0.08		
Gender	−0.66	−1.18, −0.14	−0.14	−2.52	0.01		
Age	0.27	0.03, 0.51	0.13	2.22	0.02		
SOP-Striving ^9^	−0.07	−0.17, 0.01	−0.10	−1.66	0.09		
SOP-Critical ^10^	0.05	−0.01, 0.12	0.09	1.46	0.14		
SPP ^11^	0.01	−0.03, 0.07	0.50	0.66	0.50		
DV^1^: Peer problems							
Step 1				16.84	<0.001	0.09	0.09
Constant	5.21	3.84, 6.59		7.45	<0.001		
Gender	−0.46	−0.79, −0.13	−0.14	−2.73	0.007		
Age	−0.37	−0.51, −0.22	−0.26	−5.02	<0.001		
Step 2				8.60	<0.001	0.12	0.10
Constant	4.97	3.26, 6.68		5.71	<0.001		
Gender	−0.44	−0.77, −0.10	−0.13	−2.59	0.01		
Age	−0.32	−0.47, −0.16	−0.23	−4.03	<0.001		
SOP-Striving ^9^	−0.06	−0.12, −0.006	−0.13	−2.15	0.03		
SOP-Critical ^10^	0.01	−0.03, 0.05	0.02	0.44	0.65		
SPP ^11^	0.04	0.01, 0.08	0.15	2.48	0.01		
DV^1^: Prosocial behavior							
Step 1				9.59	<0.001	0.05	0.05
Constant	5.72	4.38, 7.16		8.15	<0.001		
Gender	0.46	0.12, 0.79	0.14	2.68	0.008		
Age	0.25	0.10, 0.39	0.18	3.36	0.001		
Step 2				5.02	<0.001	0.07	0.06
Constant	4.89	3.15, 6.63		5.54	<0.001		
Gender	0.50	0.16, 0.84	0.16	2.91	0.004		
Age	0.23	0.07, 0.39	0.17	2.90	0.004		
SOP-Striving ^9^	0.06	0.005, 0.12	0.13	2.13	0.03		
SOP-Critical ^10^	0.005	−0.04, 0.05	0.01	0.20	0.83		
SPP ^11^	−0.006	−0.04, 0.03	−0.02	−0.34	0.73		
Step 3 Interactions				4.29	<0.001	0.08	0.07
Constant	5.76	4.25, 7.27		7.50	<0.001		
Gender	0.49	0.15, 0.83	0.15	2.86	0.004		
Age	0.25	0.09, 0.41	0.18	3.10	0.002		
SOP-Striving ^9^	0.008	−0.07, 0.09	0.01	0.19	0.84		
SOP-Critical ^10^	0.008	−0.03, 0.05	0.02	0.35	0.72		
SPP ^11^	−0.007	−0.04, 0.02	0.01	−0.39	0.69		
Gender x SOP-Striving	0.11	0.002, 0.21	0.16	1.99	0.04		
Age x SOP-Striving	−0.03	−0.08, 0.02	−0.02	−1.20	0.22		
DV^1^: Total difficulties							
Step 1				4.20	.01	0.02	0.02
Constant	12.73	7.56, 18.01		4.81	<0.001		
Gender	−1.83	−3.10, −0.57	−0.15	−2.85	0.005		
Age	−0.11	−0.66, 0.43	−0.02	−0.41	0.67		
Step 2				4.54	0.001	0.06	0.05
Constant	12.72	7.15, 18.29		4.49	<0.001		
Gender	−1.54	−2.81, −0.28	−0.13	−2.41	0.01		
Age	−0.12	−0.71, 0.46	−0.02	−0.41	0.67		
SOP-Striving ^9^	−0.19	−0.41, 0.03	−0.10	−1.64	0.10		
SOP-Critical ^10^	0.22	0.05, 0.39	0.16	2.59	0.01		
SPP ^11^	0.12	−0.009, 0.26	0.12	1.83	0.06		

^1.^ Dependent variable, ^2^ unstandardized regression coefficient, ^3^ confidence interval, ^4^ standardized regression coefficient, ^5^ obtained *t*-value for each predictor variable, ^6^ probability, ^7^ proportion of variance explained, ^8^ adjusted proportion of variance explained, ^9^ Self-oriented Perfectionism-Striving, ^10^ Self-oriented Perfectionism-Critical, and ^11^ Socially Prescribed Perfectionism.
